# Effectiveness of a Combined Web-Based and Ecological Momentary Intervention for Incoming First-Year University Students: Protocol for a 3-Arm Randomized Controlled Trial

**DOI:** 10.2196/10164

**Published:** 2018-05-15

**Authors:** Benjamin C Riordan, Saleh Moradi, Kate B Carey, Tamlin S Conner, Kyungho Jang, Kelly E Reid, Damian Scarf

**Affiliations:** ^1^ Department of Psychology University of Otago Dunedin New Zealand; ^2^ Center for Alcohol and Addiction Studies Department of Behavioral and Social Sciences Brown University School of Public Health Providence, RI United States

**Keywords:** alcohol drinking in college, clinical trial, smartphone, internet

## Abstract

**Background:**

Alcohol use among university students is common, and those who drink often choose to drink heavily (ie, 4 or more drinks per session for women or 5 or more for men). Web-based interventions (WBIs), in which students complete assessments and receive personalized feedback about their alcohol use, and ecological momentary interventions (EMIs), which use mobile devices as a method of delivering intervention information, are 2 methods that have had some success in reducing alcohol use among university students.

**Objective:**

The aim of this study was to investigate the effectiveness of a combined WBI and EMI intervention to reduce alcohol use among university students.

**Methods:**

The study is a 3-arm randomized controlled trial. Participants will be randomized into either a WBI+EMI condition, a WBI-only condition, or an assessment-only control. Our sample will consist of first-year university students, recruited through 5 residential colleges at the University of Otago, New Zealand. All participants will complete an online survey at baseline (ie, before Orientation Week); those in the WBI-only and WBI+EMI conditions will immediately receive personalized feedback (ie, the WBI), whereas participants in the assessment-only condition will receive no feedback. In addition, participants randomized into the WBI+EMI, but not those in the WBI-only or assessment-only groups, will receive 8 Orientation Week (2 per day on nights with large social events) and 6 academic year EMIs (delivered fortnightly). Participants in all conditions will complete brief surveys at the end of the first and second semester and report their weekend alcohol use fortnightly throughout each semester via ecological momentary assessments.

**Results:**

The primary hypothesis is that participants in the WBI+EMI group will consume significantly fewer drinks during weekends in their first semester at university compared with WBI-only and assessment-only groups. Secondary hypotheses are that, when compared with the WBI-only and assessment-only groups, the WBI+EMI group will report consuming fewer drinks during Orientation Week, report experiencing fewer negative alcohol-related consequences after first semester, and report lower Alcohol Use Disorder Identification Test-Consumption scores following their first semester.

**Conclusions:**

This study adds to a growing body of work investigating the utility of WBIs and EMIs in curbing alcohol consumption. In addition, the study will help to inform policy approaches aimed at curbing alcohol consumption and alcohol-related harm in university students.

**Trial Registration:**

Australian New Zealand Clinical Trials Registry ACTRN12618000015246; https://www.anzctr.org.au/Trial/Registration/TrialReview.aspx?id=374104&isReview=true (Archived by WebCite at http://www.webcitation.org/6z9jRLTz6)

**Registered Report Identifier:**

RR1-10.2196/10164

## Introduction

Alcohol use among university students is common, with 63% of students reporting alcohol use in the past month [1]. Of greater concern is the fact that those who drink often choose to drink heavily (ie, 4 or more drinks per session for women or 5 or more for men), with 32% of students reporting a heavy drinking session in the past 2 weeks [1]. Extensive research has highlighted that students who drink in this way experience a range of negative alcohol-related consequences, such as blackouts [2,3], risky sexual behavior [4,5], and social problems [6]. Furthermore, often due to the fact that many students live in close proximity to one another in residential colleges, students who do not themselves drink are negatively impacted by those who do (eg, unwanted sexual advances and physical aggression) [7,8].

This pattern of heavy drinking is particularly concerning for new students, many of whom increase their drinking during the transition to university [9]. Indeed, researchers tend to find that the beginning of the academic year is characterized by high levels of alcohol use [10-14]. One factor that contributes to this increase in alcohol use is Orientation Week (a.k.a., Frosh, Freshers’ Week, Introductory Week) [14-16]. Orientation Week generally precedes the start of the academic year and includes a number of university-organized social events that help first-year students form new friendships. Although the purpose of orientation is well intentioned, research on Orientation Week from one university suggests that new students double their drinking relative to a typical week before university [14], experience 5 times as many negative alcohol-related consequences [17], and pregame before attending the social events (ie, consume alcohol *before* attending the event) [18,19].

Web-based interventions (WBIs), where students are screened and provided with personalized feedback about their alcohol use, have been suggested as a potential means of reducing alcohol use and present a cost-effective way of reaching large numbers of individuals [20-24]. WBIs are effective in reducing alcohol use [25-27] and may be successful in reducing the likelihood of nondrinking students initiating drinking [28]. The effect, however, tends to be small [25,26]. For example, a large WBI implemented in 7 of 8 universities in New Zealand with 17- to 24-year-old students in the middle of first semester did not reduce the frequency with which students consumed alcohol but did result in a small reduction in the amount of alcohol consumed during a typical drinking session [29,30].

The generally small effect size reported for WBIs may be due to the fact that they rely on students remembering the intervention information. Furthermore, WBIs rely on advice given in a likely nonsocial context (eg, sitting in one’s room completing the WBI) to transfer to a social context (eg, hanging out and having drinks with friends), neglecting the fact that social factors play a strong role in students’ drinking behavior [14,17,31-35]. Ecological momentary interventions (EMIs), which use mobile devices as a method of delivering intervention information, provide a way to extend WBIs beyond the initial treatment context, not only providing individuals with reminders about the information they were given in the WBI but also a cue to apply that information in a real-world setting [36,37]. By receiving reminders close in time to the actual behavior (eg, immediately before or during a night out drinking), EMI messages can facilitate self-management of drinking in context [37].

To date, 2 pilot studies provide some support for the efficacy of EMI messages during Orientation Week [15,38]. For example, in a pilot study, first-year students were randomized into an assessment-only condition or an EMI condition. Those in the assessment-only condition reported their drinking during Orientation Week and weekly during the academic year via ecological momentary assessments (EMAs), whereas those in the EMI condition reported their drinking and received daily EMI messages during Orientation Week. The EMI messages were sent at 7:30 PM (about the time students report drinking during Orientation Week), and the content of the messages alternated between the potential health-related and social consequences of alcohol use. The initial pilot found that women (but not men) in the EMI condition consumed significantly fewer drinks than women in the assessment-only condition during both Orientation Week (17 vs 26) and weekly during the academic year (5 vs 8) [38]. Following a series of focus groups with pilot study participants, the EMI was adapted so that messages were only sent on nights with large social events and 2 messages, rather than 1, were sent on these nights. When testing these changes in a second pilot-experimental study, students attending a relatively light drinking residential college consumed significantly fewer drinks relative to an assessment-only group during both Orientation Week (10 vs 16) and a typical academic year weekend (4 vs 7). The EMI, however, had no effect on students attending a residential college with a heavier preuniversity drinking pattern during either Orientation Week (38 vs 37) or the academic year (11 vs 9) [15]. Although these preliminary findings are promising, they have only been successful at reducing lighter drinkers’ alcohol use, suggesting that an EMI alone may not be not effective for all incoming students.

Recently, researchers have shown some preliminary success when using an EMI to supplement an in-person intervention or WBI [36,39-41]. For example, Haug et al [39] found that Swiss students attending vocational training schools who received a combined WBI and EMI reduced their prevalence of heavy drinking sessions compared with an assessment-only group. Similarly, Tahaney et al [40] found that risky drinking undergraduates who received a WBI+EMI consumed fewer weekend drinks compared with those who only received a WBI-only or an assessment-only condition. Building on these earlier studies, this protocol outlines a large-scale long-term randomized controlled trial (RCT) to test a WBI+EMI intervention for incoming first-year university students.

## Methods

### Study Design

**Figure 1:**
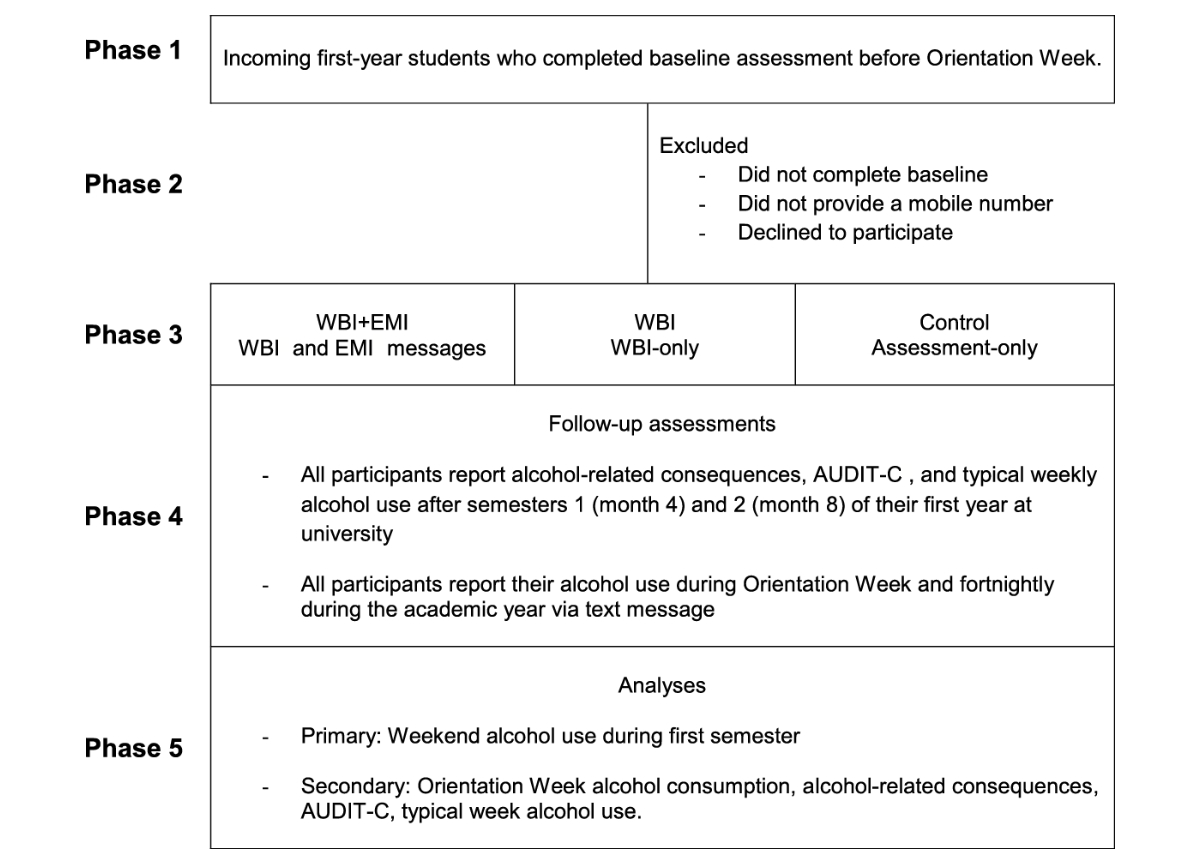
Flow of events. AUDIT-C: Alcohol Use Disorder Identification Test-Consumption; EMI: ecological momentary intervention; WBI: Web-based intervention.

The study is a 3-arm RCT. Participants will be randomized into either a WBI+EMI condition, a WBI-only condition, or an assessment-only condition (Figure 1). The WBI will be administered before the start of Orientation Week, whereas the supplemental EMI will be delivered in 2 phases: 8 messages over 4 days during Orientation Week, and 6 messages during first semester. Participants will complete surveys at baseline (before Orientation Week) and after their first (~4 months) and second semester (~8 months).

### Ethics Approval and Consent to Participate

This research was approved by the University of Otago Human Ethics Committee New Zealand. Participants were presented with the information sheet and consent form at the start of the online survey.

### Trail Status

At the time of submission, the recruitment phase had begun but not completed. Data collection for the primary outcome will be completed in June 2018. Data collection for the secondary outcomes will be completed in November 2018.

### Availability of Data and Material

All data and material supporting our findings can be obtained from the last author.

### Objectives and Hypotheses

The aim of the study was to test the effect of a WBI+EMI among incoming first-year students in New Zealand. The primary hypothesis was that participants in the WBI+EMI group will consume significantly fewer drinks during weekends in their first semester at university compared with those in WBI-only or assessment-only groups. Secondary hypotheses are that, when compared with those in the WBI-only and assessment-only groups, participants in the WBI+EMI group will report consuming fewer drinks during Orientation Week, and report experiencing fewer negative alcohol-related consequences, report lower Alcohol Use Disorder Identification Test-Consumption (AUDIT-C) scores, and less typical week alcohol use (at both 4 and 8 months follow ups). We also hypothesize that those in the WBI-only condition will consume significantly fewer drinks during weekends in their first semester at university, report consuming fewer drinks during Orientation Week, report experiencing fewer negative alcohol-related consequences, and report lower AUDIT-C scores compared with participants in the assessment-only group.

### Participants and Procedures

All incoming students who are beginning their first-year at university, are between 18 and 25 years, and living in any of the 5 residential colleges at the University of Otago will be invited to take part. The invitation email will be sent out from each of the residential colleges to their incoming cohorts. The initial email invitation will be sent 4 weeks before the first day of Orientation Week, with a follow-up reminder 2 weeks before the beginning of Orientation Week. Residential colleges will also invite students to take part by posting on their respective Facebook pages. Participants will be offered NZ $100 (US $73.12, Can $92.29, Aus $93.81, UK £51.56) remuneration for taking part in the study.

Participants who are interested in taking part will click a link to a secure webpage with information about the study and consent forms. Participants will be excluded if they decline to participate throughout the academic year or do not provide a mobile number. After completing the baseline survey (which includes a definition of a New Zealand standard drink), those who provide a mobile phone number will then be randomized into 1 of the 3 conditions (ie, WBI+EMI, WBI-only, and assessment-only). Participants randomized into the WBI+EMI condition and the WBI-only condition will automatically receive personalized feedback (ie, the WBI) based on their answers on the baseline survey. Participants randomized into the assessment-only group will not receive feedback.

Participants randomized into the WBI+EMI condition, but not those in the WBI-only condition or assessment-only condition, will receive EMIs during Orientation Week and throughout the first semester. Participants in all conditions will be asked to report their alcohol use during Orientation Week and fortnightly throughout the academic year via EMAs (ie, text messages) and complete brief surveys at the end of the first and second semester. Reimbursement will occur at the end of the academic year.

### Assessment and Outcome Measures

The primary outcome measure will be weekend alcohol use during first semester, reported via fortnightly EMAs. Secondary outcomes will include Orientation Week alcohol use, alcohol-related consequences, AUDIT-C scores, and typical weekly alcohol use (measured at baseline, after semesters 1 and 2).

#### Measures

##### Demographics

Demographic variables (age, gender, ethnicity) will be assessed at baseline.

##### Academic Year Weekend Alcohol Use

Academic year weekend alcohol use [14,15] will be assessed by fortnightly EMAs during semesters 1 and semester 2 of a students’ first year at university (“How many drinks did you have Thurs, Fri, Sat? Send reply like this: 1,5,0.”; see Table 1). This procedure has been used in prior studies with good compliance (75% completed 4 or more of the 7 academic year reports in the pilot study) [13,14].

**Table 1:** Schedule of text messages for each group during semesters 1 and 2 of the academic year.

Condition	Semester 1							Semester 2						
	Week 1	3	5	7	9	11	13	1	3	5	7	9	11	13
Control	EMA^a^	EMA	EMA	EMA	EMA	EMA	OLS	EMA	EMA	EMA	EMA	EMA	EMA	OLS^b^
WBI^c^	EMA	EMA	EMA	EMA	EMA	EMA	OLS	EMA	EMA	EMA	EMA	EMA	EMA	OLS
WBI+EMI^d^	EMA, EMI	EMA, EMI	EMA, EMI	EMA, EMI	EMA, EMI	EMA, EMI	OLS	EMA	EMA	EMA	EMA	EMA	EMA	OLS

^a^EMA: ecological momentary assessment.

^b^OLS: online survey.

^c^WBI: Web-based intervention.

^d^EMI: ecological momentary intervention.

##### Orientation Week Alcohol Use

Orientation Week alcohol use [14,15] will be assessed by 2 EMAs during Orientation Week. One message will be sent on the Thursday of Orientation Week at 2:00 PM (“How many drinks did you have Mon, Tues, Wed? Send reply like this: 1,5,0”) and the second will be sent on Sunday at 2:00 PM (“How many drinks did you have Thurs, Fri, Sat? Send reply like this: 1,5,0”). This procedure has been used in prior studies with good compliance (75% completed both reports) [13,14].

##### Negative Alcohol-Related Consequences

The number of negative alcohol-related consequences [42] experienced will be assessed by the Brief Young Adult Alcohol Consequences Questionnaire (B-YAACQ). The B-YAACQ is composed of a list of 24 alcohol consequences, and participants simply answer yes or no as to whether they have experienced each consequence in the past 3 months. The B-YAACQ will be administered at baseline and after semesters 1 and 2 (~4 and ~8 months).

##### Audit-C

The AUDIT-C [43] is composed of 3 questions and provides a brief and effective screening tool for identifying likely alcohol use disorders [43]. The AUDIT-C will be administered at baseline and after semesters 1 and 2 (~4 and ~8 months).

##### Typical Week Alcohol Use

Number of drinks consumed during a typical week will be measured retrospectively using a modified version of a timeline follow-back procedure [44]. Participants will be asked to “Think of a typical week in the last 3 months for you. Think of what you did, where you lived, what your weekly activities were. Try to accurately remember how much alcohol you typically drank.” Typical week drinking will be measured at baseline and after semesters 1 and 2 (~4 and ~8 months).

### Intervention Components

#### WBI

The WBI will provide personalized normative feedback based on the amount of alcohol participant’s report consuming during a typical week. The feedback will be specific to the University of Otago, participant’s gender, and their year at university. These specific norms will be derived from the Daily Life Study, a large study that surveyed around 2000 full-time students from the University of Otago (~10% of the university population) [31,45]. The feedback includes tailored graphics and text information regarding (1) the number of drinks consumed in the past week compared with a typical first-year student of the same gender, (2) the financial cost of drinking, (3) the number of calories consumed, and (4) the number of negative alcohol-related consequences experienced in the past 3 months compared with a first-year student of the same gender. Participants will also receive feedback on their AUDIT score, feedback on their heaviest drinking session (estimated peak Blood Alcohol Content and the effects of consuming alcohol at that level), and suggest protective behavioral strategies.

#### EMI

The EMI consists of text messages delivered during Orientation Week and fortnightly throughout the first semester. Content includes information about protective behavioral strategies, the social consequences of drinking, and campus-based social norms. The Orientation Week messages will be sent on the nights during Orientation Week historically associated with the most drinking (ie, the first-year toga party, on nights with music concerts, and the Saturday of Orientation Week). The specific content and timing of the messages are based on feedback from surveys [38], focus groups [15], and in-situ interviews with students outside Orientation Week events [18]. On days during Orientation Week with social events, participants will receive 1 message at 2:00 PM reminding them of a protective behavioral strategy mentioned in the WBI (eg, “Toga party tonight! If you are planning to have a few drinks, remember to eat. Food=energy! Eating is not cheating”). They will then receive 1 message timed to when they start drinking at 7:00 PM reminding them of the social consequences of alcohol (eg, “Remember, don’t be a dick! Your drinking can affect your mates”; Table 2 contains the complete list of Orientation Week EMIs).

During the academic year, students will receive a fortnightly social norm message tailored to their gender (eg, “Hope you had a great OWeek! The typical female scarfie drinks no more than 6 drinks per week. OWeek is a one off, now the year begins”; see complete list of text messages in Table 3).

#### Randomization

Participants will be randomized into the WBI+EMI, WBI-only, or assessment-only groups. Participants who agree to take part after the initial survey will be allocated with a number (1-6) using a random number generator. Randomization is fully computerized and, therefore, is not possible to subvert.

### Analytical Plan

#### Data Treatment

Given that our study aims to demonstrate that a combined WBI+EMI is more effective than a WBI-only and assessment-only condition, it will be categorized as a “superiority trial.” Results will be presented using both the intention-to-treat principle (ie, all participants who were assigned to a condition) and complete cases (ie, participants who completed every report) [46]. The missing data for intention-to-treat analyses will be dealt with using multiple imputation.

**Table 2:** Complete list of ecological momentary intervention text messages during Orientation Week.

#	Time	Text	Type
1.	Wed 2:00 PM	“Toga party tonight! If you are planning to have a few drinks, remember to eat. Food=energy! Eating is not cheating.”	PBS^a^
2.	Wed 6:45 PM	“These could be your friends for the year. Make sure your drinking doesn’t ruin everyone’s night.”	SC^b^
3.	Thur 2:00 PM	“Concert tonight! Remember to smash water when drinking. Subbing water while you drink will decrease hangover symptoms. OWeek is a loong week”	PBS
4.	Thur 6:45 PM	“On it? Remember to look after your friends if you are drinking!”	SC
5.	Fri 2:00 PM	“Rugby tonight! You’ve made it this far. If you’re drinking tonight, set a limit that works and stick to it!”	PBS
6.	Fri 6:45 PM	“Think about your friends if you are drinking. Don’t be the story everyone tells tomorrow.”	SC
7.	Sat 2:00 PM	“OWeek Saturday! If you’re having a wet one tonight, drink slowly. Alc can hit you like a ton of bricks!”	PBS
8.	Sat 6:45 PM	“Remember, don’t be a dick! Your drinking can affect your mates.”	SC

^a^PBS: protective behavioral strategy.

^b^SC: social consequence.

**Table 3:** Complete list of ecological momentary intervention text messages during semester 1.

Week	Text (men)	Text (women)
1	“Hope you had a great OWeek! The typical male scarfie drinks no more than 11 drinks per week. OWeek is a one off, but now the year begins!”	“Hope you had a great OWeek! The typical female scarfie drinks no more than 6 drinks per week. OWeek is a one off, now the year begins!”
3	“Drinks can set you back! The average scarfie male drinks about 11 drinks per week, that is $1144-5720 a year, OR 2-10 round trips to Raro!”	“Drinks can set you back! The average scarfie female drinks about 6 drinks a week, that is $624-3120 a year, OR 1-5 round trips to Raro!”
5	“Remember, drinks contain empty calories. The average male scarfie drinks no more than 11 drinks a week, that is about 2.3 sticks of butter”	“Remember, drinks contain empty calories. The average female scarfie drinks no more than 6 drinks a week, that is about 1.3 sticks of butter.”
7	“Hope you had a good break! During this half of the semester the typical male scarfie drinks no more than 8.6 drinks a week”	“Hope you had a good break! During this half of the semester the typical female scarfie drinks no more than 4.2 drinks a week.”
9	“This time of year, male scarfies typically drink no more than 8.6 drinks per week. That is about $894-4472 a year, OR 9-45 HUBs text books!”	“This time of year, female scarfies drink no more than 4.2 drinks per week. That is about $437-2184 per year, OR 4-21 HUBs text books!”
11	“This time of year, male scarfies drink no more than 8.6 drinks per week. That is about 1462 extra calories OR a cup of bacon fat!”	“This time of year, female scarfies drink no more than 4.2 drinks per week. That is about 714 extra calories OR half a cup of bacon fat”

#### Statistical Analysis

Given that our outcome variables of interest, with the exception of alcohol use disorders measured by AUDIT-C scale, will comprise count data (ie, number of drinks consumed and number of negative alcohol-related consequences experienced), generalized linear models (GLMs) will be employed [47]. The standard GLM, however, assumes that the observations are uncorrelated, which is certainly not the case in longitudinal designs such as this study [48,49]. Given this, extensions to the standard GLM will be employed. Briefly, generalized estimating equations (GEE) are mostly suggested for analysis of data involving 2 data levels (eg, observations clustered within individuals, or individuals clustered within groups) to investigate population-averaged changes in the outcome [50]. GLMs, however, are deemed more robust in the designs involving more than 2 levels of data (eg, observations clustered within individuals who are clustered within groups) to test individual-averaged changes in the outcome [49]. Interpreting the GEE results to make inferences about individual-specific changes over time and interpreting the GLM results to make inferences about the population’s mean change over time lead to ecological and atomistic fallacies [51].

Given our interest in both population-averaged and individual-specific changes over time, and the fact that our participants will be clustered within different colleges, we will use both GEE and GLM. Specifically, GEE will be used to investigate population-averaged changes in the primary and secondary outcomes as a result of time, type of intervention, and their interaction. To deal with the potential effect of participants nested in different residential colleges, unit dummies (ie, 1 dummy for each college except for a reference college) will be added as covariates to account for any heterogeneity between the colleges.

To investigate individual-specific changes in outcomes we will use GLM. The use of GLM allows further investigation of the residential college-specific changes in outcomes. In addition to the fixed effects of time, type of intervention, and their interaction, we will add random effects for individuals and for colleges. The addition of random effects for residential colleges is justified by our prior studies showing that different students from residential colleges have different drinking habits [15].

Prior research also suggests that alcohol interventions have different impacts on individuals with, for instance, different average alcohol consumption levels [15]. Information regarding the potential existence and the number of such unobserved, yet homogenous, subpopulations within the data, however, can only be obtained using post hoc analysis techniques such as growth mixture modeling (GMM). GMM provides the framework necessary for identifying multiple unobserved subpopulations, examining each unobserved subpopulation’s trajectory over time and in comparison with the other unobserved subpopulations [52]. For this study, GMM will shed light on possible differences in the effectiveness of the proposed intervention for students with various drinking habits.

Across analyses, baseline characteristics will be controlled for as we expect that gender and baseline alcohol consumption might influence the effectiveness of the intervention [9].

#### Sample Size Calculations

A series of power analyses were performed to estimate the sample size required to detect a small interaction effect between time and type of intervention (the estimated effect size=−0.1) on student’s number of weekend drinks. The target was at least 80% power. Given that we are only interested in 2 estimated effects (ie, the main effect intervention and the interaction effect between time and intervention), a Bonferroni-adjusted family wise alpha of .025 was assumed for the power analysis [53]. All the analyses were conducted using R (R Foundation for Statistical Computing) and were based on a dataset obtained from a previous pilot experimental study [15].

Taking a conservative approach, we first estimated the sample size for a GEE with the main effects of time, type of intervention (ie, treatment), their interaction, and the main effects of gender and baseline alcohol consumption (ie, baseline). To this end, we used the GEE and long-power packages to estimate the minimum sample size required [54,55]. The results of analysis suggested a sample size of 527 to 553 students based on assuming an unstructured or an exchangeable correlation matrix.

Next, based on Monte Carlo simulations and using the estimated sample size required for the GEE analysis (ie, 553), we evaluated the power of performing GLM analysis. All the analyses were performed using the lme4 and simr packages in R [56,57]. First, we fitted a 2-level mixed effect model with the fixed effects of time, type of intervention (ie, treatment), their interaction, gender and baseline alcohol consumption (ie, baseline), and the random effect of student. The simulation results revealed a statistical power 97%, with 95% CI=91.48-99.38. Moreover, for a 3-level mixed effect model with the fixed effects of time, type of intervention (ie, treatment), their interaction, gender and baseline alcohol consumption (ie, baseline), and the random effects of student and college, the results of simulation showed a statistical power 98%, with 95% CI=92.66-99.76. Hence, our analysis suggested that a sample of 553 students would provide sufficient power for either 2- or 3-level mixed effect models.

Finally, considering an additional 25% dropout rate [15], we came up with a final target sample size of 692 students (the pilot dataset and the details of power analyses, eg, R codes, results, and power curve plots are available upon request from the first or last author).

## Discussion

### Summary

As noted above, the transition to university is associated with an increase in alcohol consumption [9]. One factor that contributes to this increase is Orientation Week [14-16]. Indeed, research on Orientation Week suggests that new students double their drinking relative to a typical week before university [14] and experience 5 times as many negative alcohol-related consequences [17]. With the aim of curbing these increases in consumption and harm, this protocol outlines a large-scale long-term RCT to test a WBI+EMI intervention.

### Limitations

This study is not without limitations. First, although the WBI provides individually tailored feedback, the content of the EMI messages is only tailored to the student’s gender. It is, however, important to remember that EMI messages were developed based on feedback from surveys [38], focus groups [15], and in-situ interviews with students outside Orientation Week events [18]. A second limitation is that there is a high degree of contact required for the assessment-only condition. It is possible that, due to regularly being asked to report how much alcohol they have consumed, participants in the assessment-only condition will reduce their levels of alcohol consumption and harm. Typically, participants in assessment-only control conditions are assessed at baseline and then at the end of the study. In this study, the assessment-condition consists of 2 assessment messages during Orientation Week and fortnightly assessment messages during the academic year (in addition to the baseline and follow-up assessments). The benefit of this approach is that we can compare the 3 arms of this RCT with a great deal of temporal precision. The limitation of this approach is that any effects observed will likely be smaller than those observed if we simply conducted a baseline assessment and long-term follow-up.

### Conclusions

This study adds to a growing body of work investigating the utility of WBIs and EMIs in curbing alcohol consumption. In addition, the study will help to inform policy approaches aimed at curbing alcohol consumption and alcohol-related harm in university students.

## Abbreviations

AUDIT-C: Alcohol Use Disorder Identification Test-Consumption
EMA: ecological momentary assessment
EMI: ecological momentary intervention
GEE: generalized estimating equations
GLM: generalized linear models
GMM: growth mixture modeling
OLS: online survey
PBS: protective behavioral strategy
SC: social consequence
WBI: Web-based intervention
